# Organ-Resolved Metabolomics of *Cibotium barometz* Reveals Rhizome-Specific Terpenoid Lactones and Phenolic-Rich Non-Officinal Organs

**DOI:** 10.3390/metabo16090693

**Published:** 2026-09-18

**Authors:** Guole Qin, Daizu Xie, Changqin Zhou, Delong Guan, Jing Song

**Affiliations:** 1 Guangxi Key Laboratory of Sericulture Ecology and Intelligent Technology Application, Guangxi Collaborative Innovation Center of Modern Sericulture and Silk, Guangxi Colleges Universities Key Laboratory of Exploitation and Utilization of Microbial and Botanical Resources, School of Chemistry and Bioengineering, Hechi University, Hechi 546300, China; 06032@hcnu.edu.cn (G.Q.); 2023660006@hcnu.edu.cn (D.G.); 2 Hechi Forestry Research Institute, Hechi 547000, China; 15078605273@163.com; 3 Zhongnong Qiaosheng (Fangchenggang) Traditional Chinese Medicine Technology Co., Ltd., Fangchenggang 538037, China; qin.2555@163.com

**Keywords:** *Cibotium barometz*, untargeted metabolomics, organ-specific metabolite allocation, tissue specificity index, phenylpropanoid–flavonoid biosynthesis, quality control markers

## Abstract

Background: *Cibotium barometz* (L.) J. Sm. is a medicinal fern whose rhizome is the sole officinal part, while roots, stems, leaves and young shoots are discarded as agricultural residues. The chemical rationale for this restriction, and the value of the discarded organs, remain undefined. Materials and methods: Here, untargeted LC–MS/MS metabolomics (UHPLC–Orbitrap) of methanol/acetonitrile/water extracts was applied to five organs of *C. barometz* (three pooled biological replicates per organ), annotating 2765 polar-to-moderately-polar metabolites. Results: Organ identity dominated the metabolic architecture of the sampled population: the first two principal components separated all five organs, with leaf and root as the two mutually opposed metabolic extremes and stem, young shoot and rhizome forming a contiguous module. Exploratory pairwise OPLS-DA combined with FDR-controlled univariate criteria yielded 193–1223 differentially accumulated metabolites per comparison, and tissue-specificity analysis assigned 2031 metabolites (73.45%) predominantly to a single organ. Discussion: Metabolite accumulation patterns along the phenylpropanoid–flavonoid pathway was spatially partitioned, with flavan-3-ols and isoflavones accumulating in root, flavone/flavonol glycosides in leaf and predominantly hydroxycinnamic acids in rhizome and young shoot. Of 22 previously reported or candidate quality control constituents, putatively annotated terpenoid lactones were detected almost exclusively in the rhizome, whereas several phenolics showed markedly higher relative abundance in root or leaf. Conclusions: These relative-abundance data indicate that the non-officinal organs are chemically complementary potential resources; absolute quantification, bioactivity and safety evaluation are prerequisites before any medicinal valorisation.

## 1. Introduction

*Cibotium barometz* (L.) J. Sm. is a large, perennial tree fern distributed mainly in tropical and subtropical Asia and is the botanical source of *Cibotii rhizoma*, commonly known as Gouji in traditional Chinese medicine [1,2]. The dried rhizome is the sole officinal organ recognised by the Chinese Pharmacopoeia and has traditionally been prescribed to dispel wind dampness, strengthen tendons and bones and alleviate weakness or pain of the lower back and lower extremities [1,3]. The species also has considerable ethnomedicinal importance in other Asian countries, where its rhizome and golden hairs have been used for rheumatism, neuralgia, wounds and haemostasis [1,2]. However, medicinal and ornamental exploitation, together with habitat loss and slow natural regeneration, has caused substantial pressure on wild populations [4,5]. *Cibotium* species are consequently listed as Class II nationally protected wild plants in China, while *C. barometz* is regulated under Appendix II of the Convention on International Trade in Endangered Species of Wild Fauna and Flora [1,2,6]. These circumstances create an urgent need to improve resource efficiency while maintaining the pharmacognostic identity of the officinal rhizome.

Phytochemical and pharmacological investigations of *C. barometz* have nevertheless concentrated overwhelmingly on the rhizome. More than 100 constituents have been reported, including phenolic acids and their glycosides, flavonoids, lignans, steroids, fatty acids, polysaccharides, sesquiterpenes, hemiterpene glycosides and triterpenoids [7,8,9,10]. Several compounds isolated from the rhizome, including cibotiumbarosides and glycoglycerolipids, inhibit osteoclast formation, while rhizome extracts reduce ovariectomy-induced bone loss and protect cartilage through regulation of inflammatory and matrix-degradation pathways [7,8,11]. Other studies have described antioxidant, hepatoprotective and antimicrobial activities [9,12]. Transcriptomic and biochemical analyses have further identified enzymes associated with triterpene biosynthesis, and recent metabolome–transcriptome integration has demonstrated age-dependent changes in flavonoids and other secondary metabolites within the rhizome [13,14]. Collectively, these studies support the medicinal value of *Cibotii rhizoma*, but they do not establish whether its chemical profile is representative of the whole plant or explain why the rhizome, rather than another organ, should serve as the exclusive medicinal material.

Plant metabolism is intrinsically spatial. Roots, stems, leaves, meristems and storage organs differ in their developmental origin, cellular composition and ecological function, and therefore impose distinct demands on primary metabolism and specialised chemical defence [15,16,17]. Phenylpropanoids, flavonoids and terpenoids, in particular, are frequently synthesised, transported or stored in restricted cell types and organs. Consequently, the organ selected for harvesting may capture only one sector of a plant’s chemical diversity. This issue is especially important for medicinal species in which one organ is retained as the crude drug while the remaining biomass is treated as low-value agricultural residue.

LC–MS/MS-based untargeted metabolomics on high-resolution Orbitrap instruments combines broad chemical coverage with accurate-mass and MS/MS-based annotation, making it well suited to resolving such organ-level differences [18]. Comparative studies in *Polygonum cuspidatum*, *Kadsura coccinea*, *Pueraria lobata* and *Camellia luteoflora* have shown that medicinal and non-medicinal organs possess strongly differentiated metabolomes and that conventionally discarded stems, leaves or flowers may contain unique or quantitatively superior bioactive compounds [19,20,21,22]. Organ-resolved metabolomics can therefore provide both a chemical rationale for pharmacopoeial organ selection and a basis for the valorisation of underused tissues.

In the present study, untargeted LC–MS/MS metabolomics was used to construct an organ-resolved chemical atlas of *C. barometz*. To our knowledge, this is the first systematic comparison encompassing the root, stem, leaf, young shoot and rhizome of this species. We aimed to characterise organ-level differentiation of the polar and moderately polar metabolome among the five organs; identify differentially accumulated and organ-preferential metabolites; resolve the spatial partitioning of phenylpropanoid–flavonoid and other specialised metabolite pools; and compare the organ distributions of the pharmacopoeial marker protocatechuic acid and of previously reported or candidate quality control constituents. By linking organ identity with metabolite accumulation, this study seeks to clarify the chemical basis for the rhizome as the officinal organ and to evaluate, at the level of chemical composition, the reuse potential of tissues currently discarded during cultivation and processing.

## 2. Materials and Methods

### 2.1. Plant Material and Sampling

Whole plants of *Cibotium barometz* (L.) J. Sm. were collected in March 2026 from a cultivation base in Fangchenggang, Guangxi Zhuang Autonomous Region, China (ca. 21.7° N, 108.4° E). The geographical location of the sampling site is shown in Figure S1. Species identity was confirmed by Prof. Guole Qin, Hechi University, and a voucher specimen was deposited in the Herbarium of Hechi University.

Sampling followed a stratified random design within a single plot. Fifteen healthy plants of comparable size and developmental stage (3–4-year-old cultivated plants) were selected at random and excavated whole. Five organs were separated from each plant: root (WR), stem (WS, the petiole/stipe of the mature frond), leaf (WL, mature frond lamina), young shoot (WN, unexpanded crozier) and rhizome (WP). Plants were randomly assigned to three groups of 3–5 individuals; within each group, the corresponding organs of all individuals were pooled to form one biological replicate. Thus, each organ was represented by three pooled replicates (*n* = 3; 15 samples in total), the three replicates of an organ were composed of non-overlapping individuals, and all five organs of a given individual contributed to replicates with the same replicate number. Pooling was adopted to obtain sufficient homogeneous material from this protected species while minimising the number of plants destructively sampled. We note explicitly that pooling averages out inter-individual variability: *n* = 3 refers to three pools rather than 15 independent plants per organ, so the design supports comparisons among organs of the sampled population but does not allow estimation of plant-to-plant variance.

Rhizomes were freed of soil and golden trichomes; all organs were rinsed with deionised water, blotted dry, immediately frozen in liquid nitrogen in the field and transported on dry ice to the laboratory, where they were stored at −80 °C for no longer than two weeks before lyophilisation. Frozen tissues were lyophilised (Scientz-100F, Scientz, Ningbo, China), ground in a ball mill (MM 400, Retsch, Haan, Germany) at 30 Hz for 90 s and the powder was stored at −80 °C until extraction (≤2 weeks).

### 2.2. Metabolite Extraction and Untargeted LC–MS/MS Analysis

Untargeted metabolomic profiling was performed by Shanghai Personal Biotechnology Co., Ltd. (Personalbio, Shanghai, China). The five organ sample sets were extracted and acquired in the same analytical batch, and with the same standardised protein-precipitation workflow, instrumentation and processing pipeline, as the wild-collected, field-domesticated and tissue-cultured rhizome samples described in our companion study [23], so that the two datasets are directly comparable. The method description given in Section 2.2 of the originally submitted version of this manuscript was incorrect and has been corrected here; the nature of that error, and the evidence that the data reported throughout this manuscript were in fact acquired on the platform described below, are set out in the Provenance of the Data and Relationship to the Originally Submitted Method Description section. The methanol/acetonitrile/water extraction system recovers the polar-to-moderately-polar compound classes (phenolic acids, flavonoids and their glycosides, organic acids, amino acids, alkaloids, nucleosides, oxygenated terpenoids and lactones) that dominate the reported chemistry and quality control interest of *Cibotii rhizoma*. Highly lipophilic compounds (free fatty acids, fatty-acid methyl esters, phytol, caryophyllene oxide) are only partially extracted by this solvent and are ionised with low efficiency by electrospray; their relative abundances should therefore be regarded as semi-quantitative. Non-polar lipids and volatile terpenes were outside the intended scope of this study, and the dataset is described throughout as a polar/semi-polar metabolome rather than a complete metabolome.

Briefly, 100 mg of lyophilised powder was weighed into a 2 mL centrifuge tube, and 1000 µL of pre-chilled extraction solvent (methanol/acetonitrile/water, 2:2:1, *v*/*v*/*v*, −20 °C) containing internal standards was added. The internal standards were used to monitor extraction efficiency, injection reproducibility and retention-time stability and were not used for absolute quantification. Samples were vortexed for 60 s, homogenised in a ball mill at 45 Hz for 4 min and sonicated in an ice-water bath for 30 min. Proteins and macromolecules were precipitated at −20 °C for 1 h, after which samples were centrifuged at 12,000× *g* for 15 min at 4 °C. The supernatant was transferred to a fresh tube and evaporated to dryness in a vacuum concentrator. The dried residue was reconstituted in 200 µL of acetonitrile/water (1:1, *v*/*v*), vortexed, sonicated in an ice-water bath for 10 min and centrifuged again at 12,000× *g* for 15 min at 4 °C. The clarified supernatant was transferred to autosampler vials, kept at 4 °C in the autosampler and analysed within 24 h. All 15 samples were processed with the same reagent lots and in a single extraction session, so that any systematic analyte loss would be uniform rather than organ dependent.

Quality control (QC) samples were prepared by pooling equal aliquots (20 µL) of every experimental extract and were processed and analysed with the identical procedure. Solvent blanks (extraction solvent processed without tissue) were prepared in parallel to identify background and carry-over features. Chromatographic separation was performed on a Vanquish UHPLC system (Thermo Fisher Scientific, Waltham, MA, USA) fitted with an ACQUITY UPLC HSS T3 column (100 mm × 2.1 mm, 1.8 µm; Waters, Milford, MA, USA) maintained at 40 °C. The autosampler was held at 4 °C, the injection volume was 2 µL, and the flow rate was 0.3 mL min^−1^. For positive ion mode, mobile phase A was 0.1% formic acid in water and mobile phase B was 0.1% formic acid in acetonitrile; for negative ion mode, mobile phase A was 5 mM ammonium acetate in water and B was acetonitrile. The gradient was: 0–1 min, 2% B; 1–9 min, 2–98% B; 9–12 min, 98% B; 12–12.1 min, 98–2% B; 12.1–15 min, 2% B for re-equilibration.

Mass spectrometry was carried out on an Orbitrap Exploris 120 mass spectrometer (Thermo Fisher Scientific) equipped with a heated electrospray ionisation (HESI) source. The two polarities were acquired as two independent injections of every sample (separate positive-mode and negative-mode sequences) rather than by polarity switching, in order to maximise metabolome coverage. Source conditions were spray voltage +3.5 kV (positive)/−3.2 kV (negative), sheath gas 40 arbitrary units, auxiliary gas 10 arbitrary units, ion transfer tube 320 °C and vaporiser 350 °C. Acquisition used a data-dependent (full-scan–ddMS^2^, “top-N”) strategy: a full MS^1^ survey scan over m/z 70–1050 at a resolving power of 60,000 (FWHM at *m*/*z* 200), followed by higher-energy collisional dissociation MS^2^ of the most intense precursors at a resolving power of 15,000 with stepped normalised collision energies of 20, 40 and 60, an isolation window of 1.6 Da and dynamic exclusion. No predefined precursor → product transition list, no compound-specific declustering potential or collision energy, and no retention-time scheduling were used at any stage; the acquisition was therefore hypothesis free and untargeted, and compound identity was assigned only afterwards, in silico, from the accurate-mass MS^1^ and MS^2^ data (Section 2.3). Samples were injected in a randomised order to avoid batch- and run-order artefacts. The sequence was preceded by five QC conditioning injections to equilibrate the system, one QC injection was made after every three experimental samples (four QC injections in total) and solvent blanks were injected at the beginning, middle and end of the sequence. Overlaid QC total ion chromatograms are shown in Figure S1 (positive mode) and Figure S2 (negative mode), and in Table S2.

#### Provenance of the Data and Relationship to the Originally Submitted Method Description

We record explicitly, and without qualification, what happened. This work is one of two manuscripts prepared from a single set of *Cibotium barometz* samples that were submitted together to Shanghai Personal Biotechnology Co., Ltd. and analysed in one combined analytical batch: the nine rhizome samples comparing wild-collected, field-domesticated and tissue-cultured material, reported separately [23], and the fifteen organ samples reported here. Only one metabolomics experiment was ever performed on this material, on the Vanquish UHPLC–Orbitrap Exploris 120 platform described in Section 2.2. Section 2.2 of the originally submitted version of the present manuscript described a broadly targeted workflow on a SCIEX QTRAP 6500+ with scheduled multiple-reaction monitoring (MRM), a Shimadzu Nexera X2 system, an Agilent SB-C18 column, 70% aqueous methanol extraction. That description was erroneous: it was carried over from an earlier, unrelated project template during drafting and was not noticed before submission. No SCIEX QTRAP 6500+ data, no MRM data and no Metware-generated data exist for this study, and none of the numbers reported in this manuscript were ever derived from such a workflow. The revision therefore does not report a re-analysis on a different platform and does not change a single reported value: the dataset, the feature table, the annotations, the figures and every statistic in Section 3.1, Section 3.2, Section 3.3, Section 3.4, Section 3.5 and Section 3.6 are unchanged from the original submission. What changed is only that the Methods now describe the instrumentation that actually produced them. We apologise unreservedly for the error and for the reviewer’s time spent on it.

Because a reader cannot be asked to take this on trust, the deposited data were audited against the two competing method descriptions, and the audit is reproducible from the deposited files by the script provided as Supplementary Table S2. Six features of the data are diagnostic, and all six are incompatible with scheduled MRM on a triple-quadrupole/linear ion-trap instrument. (i) The alignment contains 34,294 detected features, of which only 2317 (6.8%) carry a library-matched compound name; the remaining 31,977 are unnamed accurate-mass/retention-time features with no formula and no library score. A scheduled-MRM acquisition can only return the predefined transitions it was programmed to record and cannot, by construction, generate tens of thousands of unidentified features. (ii) Features are annotated across more than forty distinct adduct species, including [M+NH_4_]^+^, [M+Na]^+^, [M+ACN+H]^+^, [M+CH_3_OH+H]^+^, [M+H−H_2_O]^+^, [2M+H]^+^, [2M+Na]^+^, [M+2H]^2+^ and [M−2H]^2−^; multiply charged and cluster adducts of this kind are assigned from full-scan isotope patterns and are not produced by a curated MRM transition list. (iii) Precursor *m*/*z* values are reported to five decimal places, and for the 1633 features whose annotation carries an unambiguous molecular formula and adduct, the measured m/z agrees with the theoretical adduct mass with a median absolute error of 1.65 ppm (69.1% within ±3 ppm, 73.6% within ±5 ppm). Sub-2-ppm mass accuracy is a property of an Orbitrap analyser and is unattainable on the unit-resolution quadrupole analyser of a QTRAP 6500+. (iv) Positive- and negative-mode features (17,957 and 16,337 respectively) form two separate, internally complete alignments, consistent with the two independent polarity injections described above. (v) All retention times fall between 0.02 and 7.99 min, with 79.0% eluting within the 1–9 min 2 → 98% B ramp of the 15 min HSS T3 gradient in Section 2.2. (vi) The processing tolerances, gap filling and adduct/isotope deconvolution reported in Section 2.3 are Compound Discoverer 3.3 parameters and have no counterpart in Analyst v1.7.

The audit also confirms that the fifteen organ samples reported here were acquired in the same batch as the nine rhizome samples of the companion study [23], which is the strongest available evidence of provenance because that dataset is already published and independently inspectable. The deposited annotation table is a single alignment carrying all 24 biological sample columns (WR, WS, WL, WN, WP, DM and ZP) plus the four pooled QC injections, with an identical feature index and identical annotation record across all of them; two separately acquired experiments cannot share one alignment. Recomputing group abundances from the WP, DM and ZP columns of this table reproduces the published composition of that study, including citric acid as the dominant annotated constituent of WP and ZP but not of DM, the prominence of taxuspine C and L-glutamine in DM, and of gluconic acid and L-arginine in ZP. Finally, the pooled QC is demonstrably a pool of the combined batch rather than of the organ samples alone: the QC profile correlates more closely with the mean of all 21 biological samples (r = 0.927) than with the mean of the 15 organ samples on their own (r = 0.882), which is expected only if the rhizome samples contributed to the same QC pool. The four QC injections were mutually near identical (pairwise r = 0.997; median per-feature QC RSD 3.2%), confirming that the single combined sequence was analytically stable throughout. A summary of the complete audit is given in Table S3, and the raw instrument files and the Compound Discoverer 3.3 result files underlying it are available as stated in the Data Availability Statement.

### 2.3. Data Pre-Processing, Quality Assessment and Unsupervised Analysis

A standard data process procedure was adopted [24]. Raw files were processed in Compound Discoverer 3.3 (Thermo Fisher Scientific) for peak detection, retention-time (RT) alignment, deconvolution, gap filling and area integration. Processing tolerances were as follows: precursor mass tolerance 5 ppm, fragment mass tolerance 10 ppm, RT alignment window 0.2 min, signal-to-noise threshold 3, minimum peak intensity 1 × 10^5^.

System stability was assessed before downstream analysis by visual inspection of the overlaid QC TICs for retention-time and intensity reproducibility, evaluation of signal drift across the analytical sequence, monitoring of internal-standard RT and peak-area drift and calculation of the relative standard deviation (RSD) of each feature across QC injections. Features with QC RSD > 30% were discarded, and unsupervised PCA was used to confirm tight clustering of QC injections relative to the biological groups. QC performance is summarised quantitatively in Section 3.1 and Table S2 (per-feature QC RSD distribution, internal-standard RT and area drift, and the number of features removed by the RSD filter).

Annotation was performed by matching accurate precursor mass, isotope pattern and experimental MS/MS spectra against the Personalbio in-house authentic-standard library and the public libraries mzCloud, HMDB, KEGG, MassBank and METLIN. Acceptance criteria were precursor mass error ≤ 5 ppm, isotope-pattern fit ≥ 70%, and library spectral match score ≥ 80 (best match, forward search). Identification confidence was assigned according to the Metabolomics Standards Initiative (MSI): level 1, RT and MS/MS matched to an authentic standard analysed on the same platform; level 2, MS/MS spectral match to a library without a co-analysed standard; level 3, compound class assigned from accurate mass and characteristic fragments only. Because the great majority of annotations are at levels 2–3, isomeric ambiguity cannot be excluded, and all compound names should be read as putative unless explicitly stated otherwise; in particular, the rhizome-associated terpenoid lactones discussed in Section 3.6 (michelenolide, corialactone B, scortechinone P and related structures) are level 2/3 annotations that have not been confirmed with reference standards. Table S1 reports, for every feature, the experimental MS1 *m*/*z*, mass error (ppm), ionisation mode, adduct, RT in a representative QC injection, library source of the match, spectral match score, MSI level, assigned chemical class and the raw and normalised peak area in each of the 15 samples plus the QC injections. Metabolites were assigned to chemical classes from the library annotation together with KEGG compound classification.

### 2.4. Supervised Discrimination and Identification of Differentially Accumulated Metabolites

OPLS-DA [25] was applied to each of the ten pairwise organ comparisons as an exploratory, supervised complement to PCA, primarily to derive variable importance in projection (VIP) values for metabolite prioritisation. Models (one predictive plus one orthogonal component; ropls 1.32.0, log-transformed, Pareto-scaled data) were evaluated by leave-one-out cross-validation (R^2^Y, Q^2^) and by exhaustive label permutation. Because each comparison contains only six observations, exactly C(6,3) = 20 distinct label assignments exist, so the minimum attainable permutation *p*-value is 0.05; models were retained when Q^2^ > 0.5 and the true-label Q^2^ exceeded all 19 alternative permutations (*p* = 0.05). Given the small group size relative to the number of variables, near-perfect R^2^Y values are expected and were not interpreted as evidence of model validity; OPLS-DA outputs were therefore used only for ranking metabolites, and no biological conclusion rests on the discriminant models alone. Fold changes were calculated from organ mean abundances, and significance was assessed by two-tailed Student’s *t*-tests on log-transformed abundances with Benjamini–Hochberg (BH) control of the false discovery rate (FDR). With *n* = 3 per group, distributional assumptions cannot be verified for individual metabolites; the *t*-test was therefore used as one element of a composite screening criterion rather than as a stand-alone inferential test, and metabolites satisfying VIP ≥ 1, |log_2_ fold change| ≥ 1 and FDR < 0.05 were defined as differentially accumulated metabolites (DAMs).

### 2.5. Tissue-Specificity Analysis

Organ preference was quantified with the tissue-specificity index Tau [26], τ = Σ_i_(1 − x_i_)/(N − 1), where N = 5 organs and x_i_ is the mean abundance in organ i divided by the maximum organ mean. Organ means were computed on the linear (untransformed) scale after missing-value imputation as described in Section 2.3; τ ranges from 0 (uniform) to 1 (exclusive to one organ). Metabolites with τ > 0.6 were classified as preferentially accumulated (“organ-specific” hereafter denotes predominant, not exclusive, accumulation) and assigned to the organ of maximal mean abundance. Counts, medians and ranges of τ were compared among organs and displayed as beeswarm plots, and the metabolites with the highest τ per organ were visualised across all 15 samples as a heatmap of per-metabolite z-scores of log_2_ abundance (colour range −2 to +2).

### 2.6. Pathway Analysis, Marker Profiling and Statistics

Annotated metabolites were mapped to KEGG compound identifiers and pathways [27]. Over-representation of the DAM set of each pairwise comparison was tested against 187 pathways containing 3–300 detected compounds using the hypergeometric test, with all detected metabolites as background and BH correction within each comparison. Because no pathway reached FDR < 0.05 (Section 3.5), enrichment results are presented and interpreted throughout as exploratory trends, not as statistically supported enrichment. Metabolites annotated to phenylpropanoid, flavone and flavonol, flavonoid, isoflavonoid and stilbenoid/diarylheptanoid/gingerol biosynthesis were displayed as a heatmap of per-metabolite z-scores of organ mean abundance [13,28,29]. Pathway “activity” was defined descriptively, for each of the 155 pathways with ≥5 detected members, as the mean member z-score in a given organ; this is a summary of steady-state pool sizes and does not represent metabolic flux. Organ-specific metabolites (τ > 0.6) were linked to the twelve pathways with the highest activity scores in a Sankey diagram (ggalluvial 0.12.5) [30].

Twenty-two constituents were profiled as quality control (QC) markers. Throughout this work, “pharmacopoeial marker” refers only to protocatechuic acid, the quantitative marker specified by the Chinese Pharmacopoeia; the remaining 21 compounds are previously reported constituents of C. barometz or candidate markers proposed in the literature. All 22 were retrieved from the untargeted dataset by name, synonym and KEGG identifier matching; no separate targeted or MRM panel was acquired for them. Organ-defining metabolites with unusual annotations (e.g., 8-anilino-1-naphthalenesulfonic acid, S-(−)-spirobrassinin, penniclavine) were manually re-examined for precursor/product ion consistency, retention time and MS/MS match score; three features that failed this audit were excluded from fingerprint lists and are flagged in Table S1.

Differences among the five organs for nine representative phenolic markers were tested on log_2_-transformed intensities. Normality (Shapiro–Wilk) and homogeneity of variance (Levene’s test) were assessed; because variance heterogeneity could not be excluded, Welch’s ANOVA was used as the primary test with BH correction across the nine markers, followed by Games–Howell post hoc pairwise comparisons, and the Kruskal–Wallis test was run as a non-parametric sensitivity check. A marker was assigned a dominant organ only when the Welch ANOVA [31] was significant after FDR correction (FDR < 0.05) and the Games–Howell test [32] separated the maximal organ from at least three of the four others; otherwise, it was classified as not organ discriminating.

### 2.7. Data Processing and Visualisation

Normalised peak areas exported from Compound Discoverer were used as relative abundances; no additional internal-standard correction was applied. Missing values were replaced by half of the minimum positive value of the corresponding metabolite, and the matrix was log_2_-transformed. Analytical and biological reproducibility was evaluated by Spearman rank correlation between all 15 samples, visualised as a clustered heatmap with organ annotation bars (pheatmap 1.0.12), and by the coefficient of variation (CV = SD/mean) of each metabolite within each organ, computed on untransformed abundances and displayed as cumulative distribution curves with the fraction of metabolites below CV = 30% and the median CV reported per organ.

Principal component analysis (PCA) was performed on the log-transformed, unit-variance-scaled matrix of all retained metabolites (prcomp), with the pooled QC injections projected into the same space to verify analytical stability; scores of the first two components were plotted with 95% confidence ellipses, and variance explained by the first five components was recorded. Global inter-organ relationships were additionally quantified by Pearson correlation between organ mean profiles.

Peak detection, QC evaluation, PCA and differential screening were performed on the Genes Cloud platform (Shanghai Personal Biotechnology Co., Ltd., Shanghai, China; Tau calculation, distance and correlation analyses, statistical testing and figure preparation used custom R scripts (R v4.3.1). Unless stated otherwise, data are mean ± standard deviation of three pooled biological replicates.

## 3. Results

### 3.1. Overview of the Metabolite Landscape Across Five Organs and Data Quality Assessment

**Figure 1:**
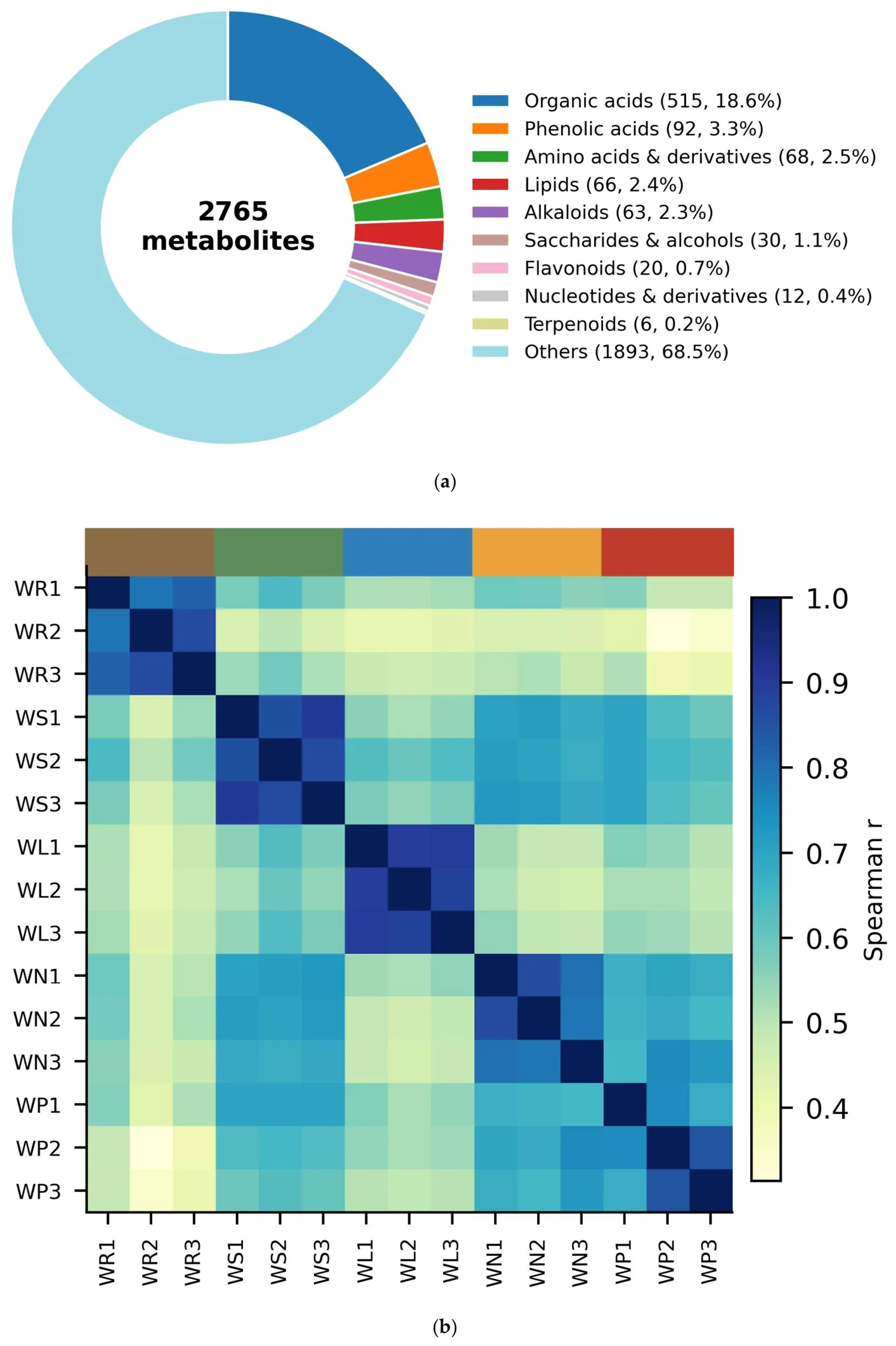

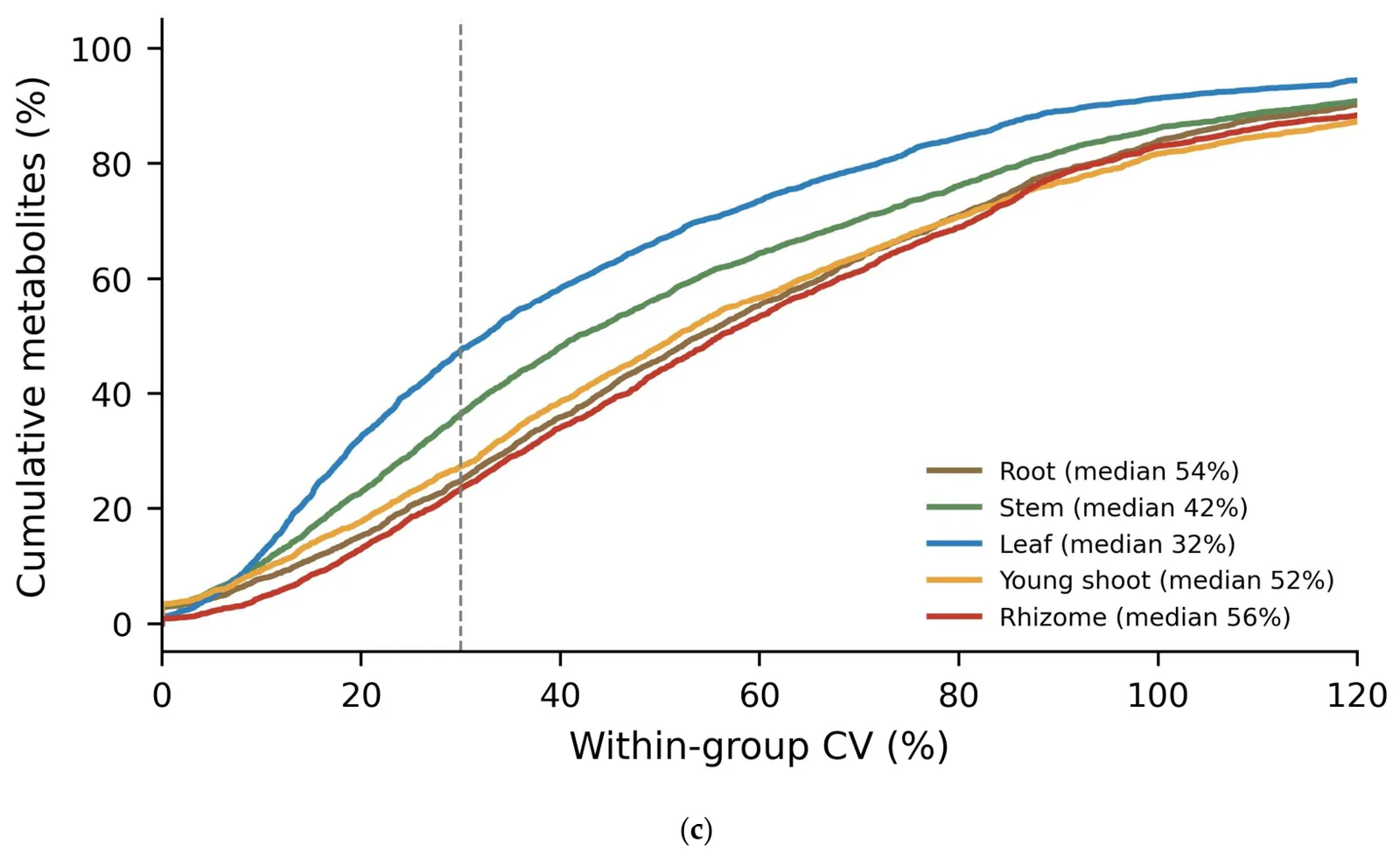
Composition and reproducibility of the metabolome across five organs. (**a**) Doughnut chart showing the classification of the 2765 metabolites detected into ten chemical classes; the number and percentage of metabolites in each class are given in the legend. (**b**) Heatmap of pairwise Spearman correlation coefficients among all 15 samples. Colour bars above the heatmap indicate organ of origin (WR, root; WS, stem; WL, leaf; WN, young shoot; WP, rhizome); numbers 1–3 denote biological replicates. (**c**) Cumulative distribution of within-group coefficients of variation (CV) for each organ. The dashed vertical line marks the CV = 30% threshold; median CV values are given in the legend.

Untargeted metabolomic profiling of five organs (root, stem, leaf, young shoot and rhizome; *n* = 3 biological replicates per organ, 15 samples in total) yielded a total of 2765 annotated metabolites, which were assigned to ten chemical classes (Figure 1a). Organic acids constituted by far the largest annotated class (515 compounds, 18.6% of all metabolites), followed by phenolic acids (92, 3.3%). Flavonoids (20, 0.7%) and terpenoids (6, 0.2%) accounted for smaller fractions, whereas the remaining 1893 compounds (68.5%) could not be assigned to these primary classes and were grouped as “others”. Although flavonoids and terpenoids were numerically scarce, both classes are of particular pharmacological interest, and their detection provided a basis for the organ-resolved analyses described below.

Pairwise Spearman correlation analysis of all 15 samples revealed coefficients ranging from 0.314 to 0.899 (mean *r* = 0.598, median *r* = 0.574; Figure 1b). Replicates from the same organ consistently formed high-correlation blocks along the diagonal, confirming acceptable reproducibility of sample collection, extraction and detection. Notably, the correlation structure was itself informative: young shoot and rhizome samples showed substantial cross-organ similarity, whereas root and leaf samples were comparatively isolated from all other organs, indicating that between-organ chemical divergence exceeded within-organ technical and biological variation.

Within-group variability was further quantified by the coefficient of variation (CV) of each metabolite in each organ (Figure 1c). Leaf displayed the most stable profile (median CV = 32.5%), followed by stem (41.8%), whereas young shoot (51.7%), root (54.1%) and rhizome (56.1%) exhibited greater heterogeneity, with fewer than 30% of metabolites below CV = 30% in these three organs. This dispersion partly reflects biological pooling variance and possibly the heterogeneous cellular composition of underground and meristematic tissues, although the latter remains a hypothesis not directly tested here. High within-group CVs reduce power and inflate uncertainty for individual metabolites in root, rhizome and young shoot; consequently, all organ-specificity claims in this study rest on the convergence of several independent criteria (multivariate ordination, FDR-controlled univariate testing and Tau), and single-metabolite differences involving these organs—particularly those of moderate effect size—should be interpreted with caution.

Consistency of the analytical platform was verified before interpretation. The alignment underlying this study comprises 34,294 detected features, of which 2317 carry a library-matched name in the annotation-metadata export and the remainder are unannotated accurate-mass features; the curated quantitative matrix used for all analyses below (Table S1) reports the 2765 compounds that passed the annotation criteria of Section 2.3, and Table S2 provides the corresponding per-feature annotation record (formula, adduct, ionisation mode, measured *m*/*z*, retention time and library score) for those compounds for which a complete record is available. Measured precursor masses agreed with theoretical adduct masses to a median absolute error of 1.65 ppm, 79.0% of features eluted within the main 1–9 min gradient ramp, and the four pooled QC injections were highly reproducible (pairwise r = 0.997; median per-feature QC RSD 3.2%, all retained features below the 30% cut-off of Section 2.3). These values are those expected of the UHPLC–Orbitrap data-dependent workflow of Section 2.2 and are reported in full, together with the diagnostic tests distinguishing that workflow from a scheduled-MRM acquisition, in the Provenance of the Data and Relationship to the Originally Submitted Method Description section and Table S3.

### 3.2. Organ Identity Is the Dominant Source of Metabolic Variation

**Figure 2:**
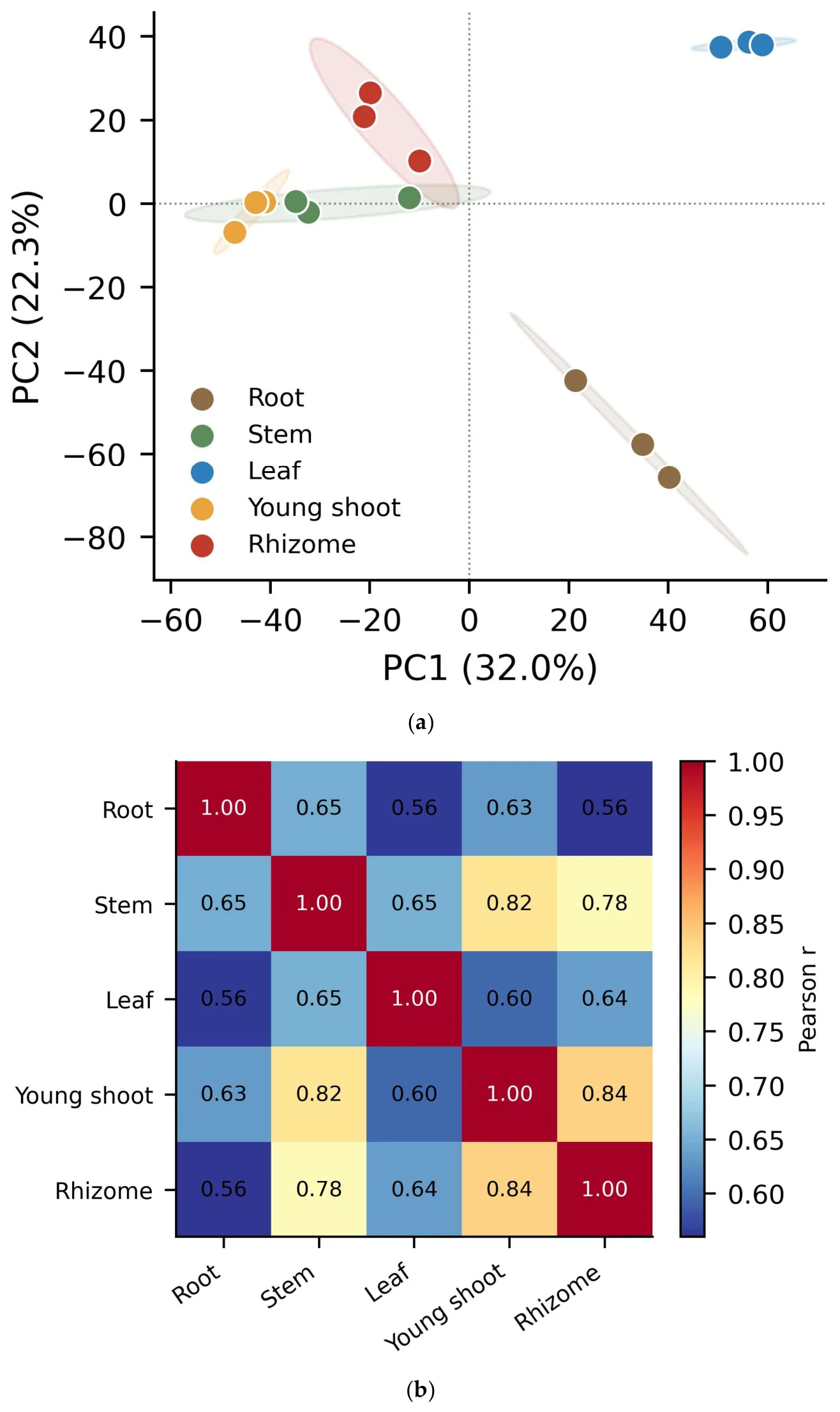
Global metabolic differentiation among organs. (**a**) PCA score plot of the 15 samples based on all 2765 metabolites. Points are coloured by organ; shaded ellipses denote 95% confidence regions for each organ. The percentage of variance explained is indicated on each axis (PC1 = 32.0%, PC2 = 22.3%; the first five components explain 78.93% of the total variance cumulatively). (**b**) Heatmap of Pearson correlation coefficients calculated between organ mean metabolite profiles. Cell values indicate the correlation coefficient; the colour scale is shown on the right.

The global structure of the dataset was initially accessed. A principal component analysis (PCA) was performed on the log-transformed and scaled abundance matrix of all 2765 metabolites. The first five principal components jointly explained 78.93% of the total variance, with PC1, PC2 and PC3 accounting for 32.0%, 22.3% and 11.6%, respectively. In the PC1–PC2 score plot, replicates of each organ clustered tightly and the five organs occupied distinct regions of the ordination space (Figure 2a). PC1 primarily separated the aerial and above-ground vegetative tissues (young shoot and stem, at negative scores) from root and leaf (at positive scores), whereas PC2 discriminated leaf (strongly positive) from root (strongly negative), placing stem, young shoot and rhizome at intermediate positions. Root and leaf were thus the two most extreme, mutually opposed metabolic states, while stem, young shoot and rhizome formed a comparatively contiguous intermediate group. The clear separation of all five organs along the first two components, together with the compactness of the replicate clusters, indicates that organ identity, rather than inter-individual variation, is the dominant determinant of metabolite composition in this species.

Correlation analysis based on organ mean profiles supported the same conclusion (Figure 2b). Pearson correlation coefficients between organs ranged from 0.560 to 0.837 (mean *r* = 0.673). The highest similarity was observed between young shoot and rhizome (*r* = 0.836), followed by stem–young shoot (*r* = 0.82) and stem–rhizome (*r* = 0.78), defining a coherent “stem–young shoot–rhizome” metabolic module. In contrast, root was the most divergent organ, showing its lowest correlations with rhizome (*r* = 0.559) and leaf (*r* = 0.56) and only moderate similarity to stem (*r* = 0.65) and young shoot (*r* = 0.63). Leaf was likewise weakly correlated with all other organs (*r* = 0.56–0.65). The clear separation of all five organs along the first two components, together with the compactness of the replicate clusters, indicates that organ identity is the dominant determinant of metabolite composition in the sampled population. Because each replicate is a pool of several plants, this statement concerns the variance among pooled organ samples; inter-individual variation was averaged within pools and was not quantified.

### 3.3. Pairwise OPLS-DA Modelling and Identification of Differentially Accumulated Metabolites

**Figure 3:**
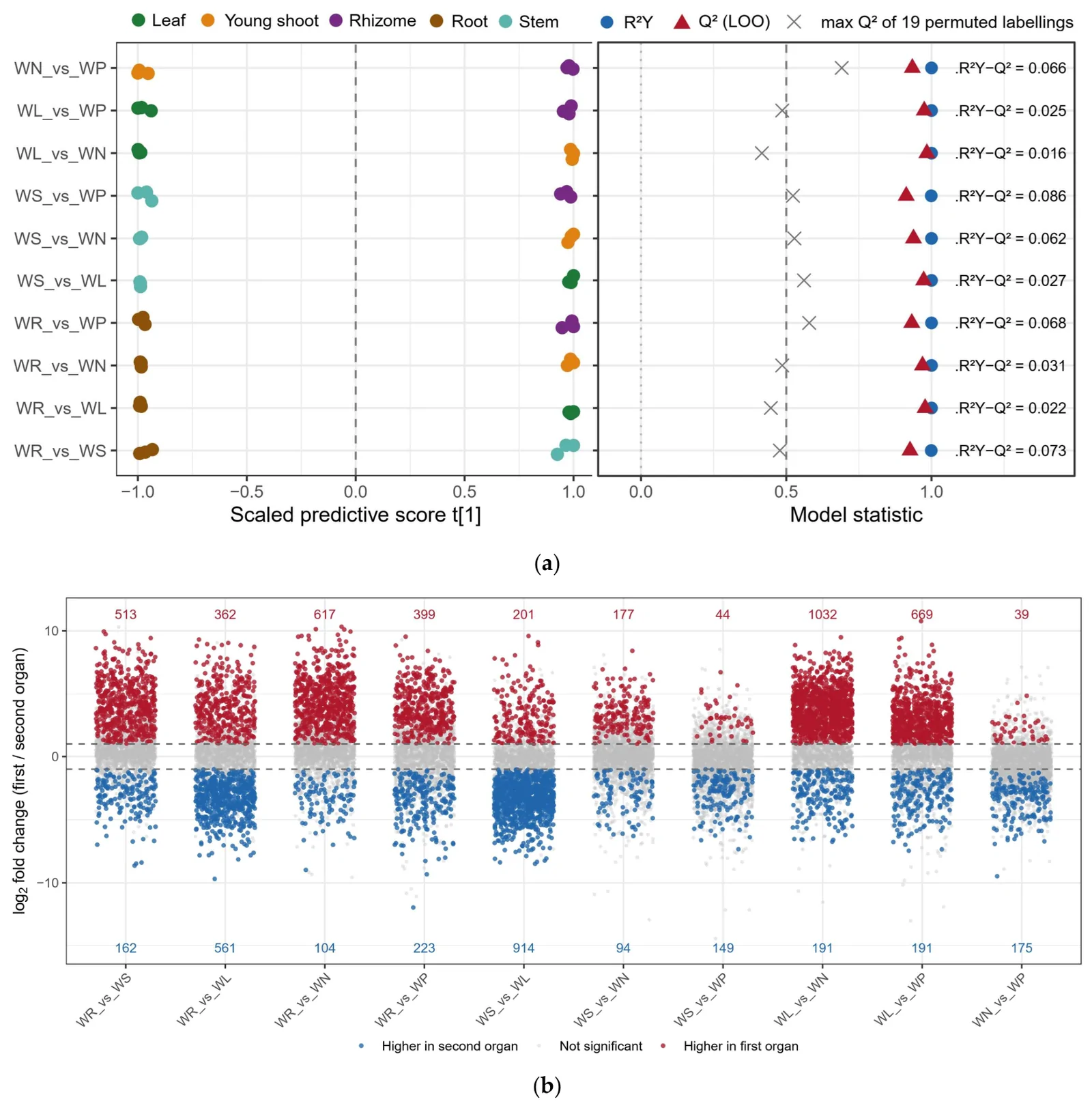
Supervised discrimination and differentially accumulated metabolites among organ pairs. (**a**) Summary of the ten pairwise OPLS-DA models (one predictive + one orthogonal component, Pareto scaling, leave-one-out cross-validation). Left: scaled predictive scores t[1] of individual samples (t[1]/max|t[1]|), coloured by organ; the first-named organ of each comparison is plotted on the negative side and the dashed line marks t[1] = 0. Right: R^2^Y (blue circles), cross-validated Q^2^ (red triangles) and the maximum Q^2^ obtained among the 19 alternative label assignments of the exhaustive permutation test (grey crosses); the dashed line indicates Q^2^ = 0.5. The R^2^Y − Q^2^ difference and the exhaustive-permutation *p*-value (minimum attainable = 0.05) are annotated for each model. (**b**) Distribution of log_2_ fold changes (first organ/second organ) for all detected metabolites in each comparison. Metabolites satisfying VIP ≥ 1, |log_2_FC| ≥ 1 and FDR < 0.05 (two-tailed Student’s t-test, Benjamini–Hochberg) are highlighted in red (higher in the first-named organ) or blue (higher in the second-named organ); other metabolites are grey. Dashed lines mark log_2_FC = ±1. Numbers above and below each column give the counts of red and blue DAMs, respectively.

OPLS-DA was applied to each of the ten pairwise organ comparisons as an exploratory, supervised complement to PCA. In every model, the two organs were separated along the predictive component t[1] (Figure 3a, left). Leave-one-out cross-validation gave Q^2^ = 0.913–0.984 and R^2^Y = 0.999–1.000 (R^2^Y − Q^2^ = 0.016–0.086), and all ten models satisfied the pre-specified Q^2^ > 0.5 criterion. Because each comparison contains only six observations, exactly 20 label assignments (10 distinct partitions) exist; exhaustive permutation showed that in all ten comparisons the true partition yielded the highest Q^2^, with the best of the nine alternative partitions reaching Q^2^ of only 0.416–0.691 (permutation *p* = 0.10, the minimum attainable value; Figure 3a, right). We stress that with six observations and 2765 variables, near-perfect R^2^Y and high Q^2^ are expected even for weakly structured data, and the exhaustive permutation can at best exclude the nine alternative partitions; these statistics therefore do not establish that the models are free of overfitting. The models were accordingly used solely to derive VIP values for metabolite prioritisation, and all organ-level conclusions rest on the convergence of PCA, FDR-controlled univariate testing and Tau.

Differentially accumulated metabolites (DAMs) were defined by the composite criterion VIP ≥ 1, |log_2_ fold change| ≥ 1 and FDR < 0.05 (Figure 3b). Across the ten comparisons, the number of DAMs varied 6.3-fold, from 193 (6.98% of all detected metabolites) between stem and rhizome to 1223 (44.23%) between leaf and young shoot. Comparisons involving leaf yielded the largest DAM sets: leaf versus young shoot (1223; 1032 higher in leaf, 191 higher in young shoot), stem versus leaf (1115; 201 higher in stem, 914 higher in leaf) and root versus leaf (923; 362 higher in root, 561 higher in leaf). Root-based comparisons were also strongly differentiated: root versus young shoot (721; 617 higher in root, 104 higher in young shoot), root versus stem (675; 513 higher in root, 162 higher in stem) and root versus rhizome (622; 399 higher in root, 223 higher in rhizome). By contrast, the three comparisons within the stem–young shoot–rhizome module produced the fewest DAMs (stem versus young shoot (271; 177 higher in stem, 94 higher in young shoot); young shoot versus rhizome (214; 39 higher in young shoot, 175 higher in rhizome); stem versus rhizome (193; 44 higher in stem, 149 higher in rhizome), in agreement with their high mutual correlation (Figure 2b). The DAM counts therefore recapitulate the ordination structure, identifying leaf and root as the two metabolically most specialised organs. Given *n* = 3 per organ and the high within-group CVs of root, rhizome and young shoot (Figure 1c), individual DAMs of moderate effect size should be regarded as candidates for confirmation rather than established differences.

### 3.4. Organ-Specific Metabolite Repertoires Revealed by Tissue-Specificity (Tau) Analysis

**Figure 4:**
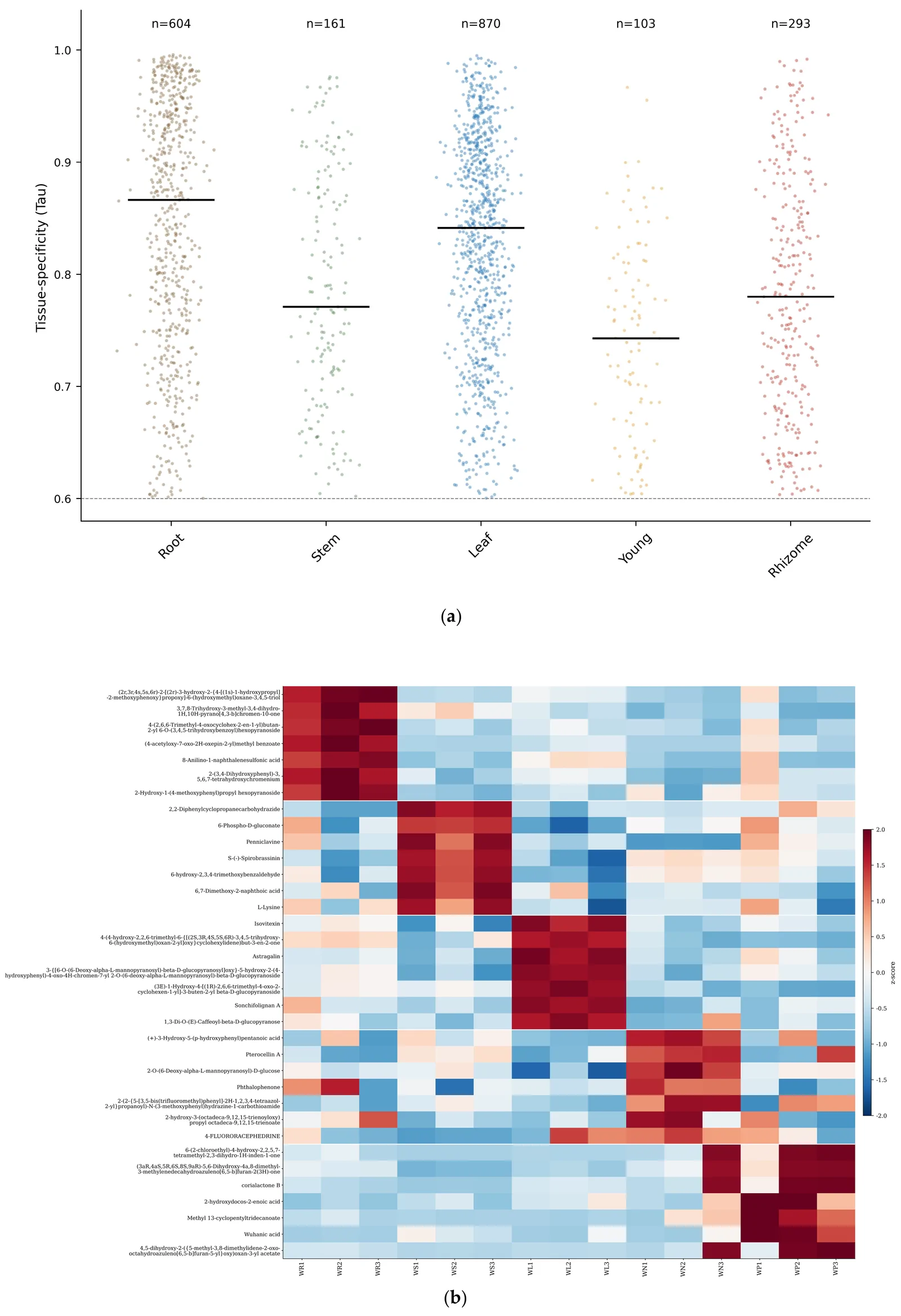
Tissue specificity of the *C. barometz* metabolome. (**a**) Beeswarm plot of tissue-specificity indices (Tau) for the 2031 organ-specific metabolites (Tau > 0.6; 73.45% of all detected metabolites), grouped by the organ of maximal abundance. Each point is one metabolite; horizontal bars denote group medians; the dashed line marks the Tau = 0.6 cut-off. Sample sizes are given above each group (root *n* = 604, stem *n* = 161, leaf *n* = 870, young shoot *n* = 103, rhizome *n* = 293). (**b**) Heatmap of the top organ-specific metabolites (highest Tau per organ) across all 15 samples (Organ-specific metabolites, Tau > 0.6) (WR, root; WS, stem; WL, leaf; WN, young shoot; WP, rhizome; three biological replicates each). Values are per-metabolite z-scores of log-transformed abundance, colour-scaled from −2 (blue) to +2 (red); rows are ordered by assigned organ.

A tissue-specificity index (Tau) was computed for every detected feature from the organ-averaged abundance profiles, and metabolites with Tau > 0.6 were assigned to the organ in which they reached maximal abundance. Of all detected metabolites, 2031 (73.45%) exceeded this threshold, indicating that the majority of the metabolome is deployed in an organ-restricted rather than a constitutive manner (Figure 4a).

The organ-specific pools were markedly unequal in size. Leaf harboured the largest repertoire (*n* = 870; median Tau = 0.841, range 0.601–0.995), followed by root (*n* = 604; median Tau = 0.866, range 0.600–0.996) and rhizome (*n* = 293; median Tau = 0.780, range 0.604–0.992), whereas stem (*n* = 161; median Tau = 0.771) and young shoot (*n* = 103; median Tau = 0.743) contributed the smallest pools. Notably, root-specific metabolites showed the highest median Tau of all organs despite being fewer than the leaf set, indicating that root-restricted compounds are, on average, the most exclusively partitioned; conversely, young-shoot and stem markers displayed both the lowest counts and the lowest median Tau values, consistent with these organs functioning largely as metabolic conduits whose chemistry overlaps extensively with adjacent tissues. Nevertheless, all five organs contained metabolites with Tau > 0.96, demonstrating that even the least specialised organs retain a small set of near-exclusive chemical signatures.

Hierarchical visualisation of the most specific metabolites (top-ranked by Tau within each organ) across all 15 individual samples confirmed that organ identity, rather than biological replicate, dominated the abundance structure (Figure 4b). Replicates of each organ formed tight, mutually exclusive high-abundance blocks: WR1–WR3 were defined by a block including 2-(3,4-dihydroxyphenyl)-3,5,6,7-tetrahydroxy flavonoid derivatives and 3,7,8-trihydroxy-3-methyl-3,4-dihydro compounds; WS1–WS3 by 6-phospho-D-gluconate, L-lysine, penniclavine and S-(−)-spirobrassinin; WL1–WL3 by the flavone C-/O-glycosides isovitexin and astragalin together with caffeoyl-glucose conjugates and sonchifolignan A; WN1–WN3 by pterocellin A, phthalophenone and oxylipin-related species; and WP1–WP3 by the terpenoid/lactone group comprising corialactone B, (3aR,4aS,5R,6S,8S,9aR)-5,6-dihydro derivatives and wuhanic acid. The near-binary contrast between each organ block and the remaining samples provides a visual corroboration of the Tau statistics and defines candidate chemical fingerprints for organ authentication.

### 3.5. Pathway Enrichment of Differential Metabolites and Organ Partitioning of Phenylpropanoid–Flavonoid Biosynthesis

**Figure 5:**
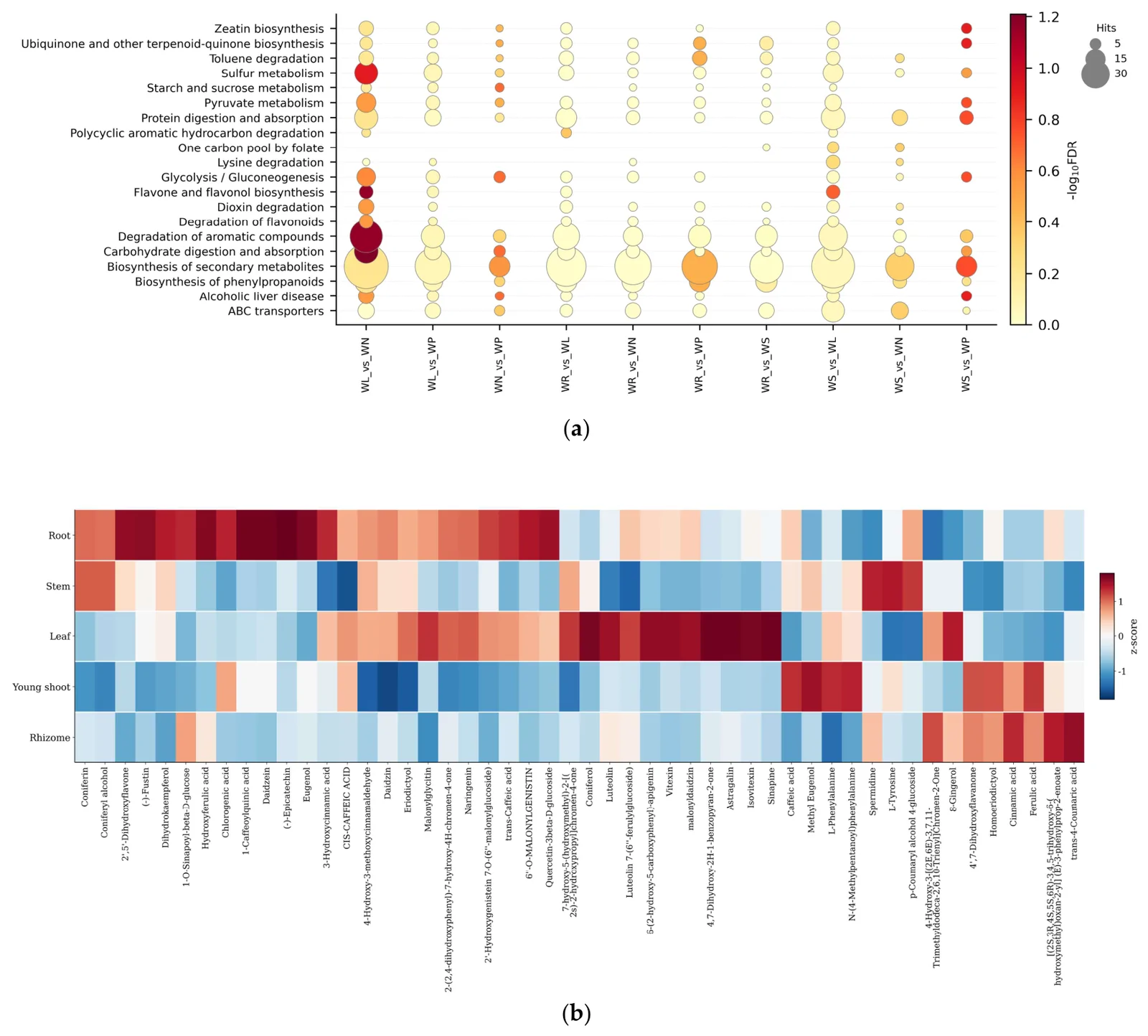
KEGG pathway enrichment and organ partitioning of phenylpropanoid–flavonoid metabolism. (**a**) Bubble plot of hypergeometric pathway enrichment for the differential metabolites of ten pairwise organ comparisons (*x*-axis) against 187 KEGG pathways with 3 ≤ size ≤ 300; the 20 pathways with the highest overall signal are shown. Bubble size denotes the number of differential metabolites mapped to the pathway (Hits); colour denotes −log_10_FDR (Benjamini–Hochberg). No pathway–comparison pair reached FDR < 0.05. Organ codes: WR, root; WS, stem; WL, leaf; WN, young shoot; WP, rhizome. (**b**) Heatmap of the 50 detected metabolites annotated to phenylpropanoid, flavone and flavonol, flavonoid, isoflavonoid, and stilbenoid/diarylheptanoid/gingerol biosynthesis across the five organs. Colour represents the per-metabolite z-score of organ-mean abundance (blue, depleted; red, enriched).

In a functional context, the ten sets of differential metabolites derived from all pairwise organ comparisons were tested against 187 KEGG pathways (3 ≤ pathway size ≤ 300) by hypergeometric enrichment (Figure 5a). After Benjamini–Hochberg correction, no pathway–comparison pair reached FDR < 0.05; all pathway-level observations below are therefore exploratory trends based on unadjusted hit counts and should not be read as statistically supported enrichment. With this caveat, the unadjusted landscape was structured. “Biosynthesis of secondary metabolites” recorded the largest hit counts in every comparison (up to >30 metabolites), confirming specialised metabolism as the principal axis of inter-organ variation. The strongest signals concentrated in the leaf-versus-young-shoot contrast (WL_vs_WN), where degradation of aromatic compounds, sulfur metabolism, flavone and flavonol biosynthesis, glycolysis/gluconeogenesis and pyruvate metabolism all attained the highest −log_10_FDR values of the dataset. Additional foci included flavone and flavonol biosynthesis in WS_vs_WL, ubiquinone/terpenoid-quinone and toluene degradation in WR_vs_WP, and zeatin biosynthesis, protein digestion and absorption, glycolysis/gluconeogenesis and pyruvate metabolism in WS_vs_WP. Together, these patterns suggest, as a hypothesis for targeted follow-up, that the aerial organs differ mainly in flavonoid and central carbon metabolite pools, whereas the underground organs differ from the shoot system in quinone/terpenoid and aromatic-compound pools.

Because phenylpropanoid-derived compounds dominated both the enrichment landscape and the pharmacopoeial interest in this species, the 50 detected metabolites annotated to phenylpropanoid, flavone/flavonol, flavonoid, isoflavonoid and stilbenoid/diarylheptanoid/gingerol biosynthesis were examined at organ resolution (Figure 5b). Root displayed the highest overall pathway activity (mean z = 0.479, median z = 0.571) with 17 metabolites strongly up-accumulated (z > 1) and (−)-epicatechin as the peak compound (z = 1.771); the root block also encompassed the upstream and flavan-3-ol/isoflavone branches, including coniferin, coniferyl alcohol, 2′,5′-dihydroxyflavone, (−)-fustin, dihydrokaempferol, 1-O-sinapoyl-β-D-glucose, hydroxyferulic acid, chlorogenic acid, 1-caffeoylquinic acid, daidzein and eugenol. Leaf ranked second (mean z = 0.311, median z = 0.486; 15 metabolites with z > 1) and was defined by a distinct downstream flavone/flavonol-glycoside module comprising luteolin, luteolin 7-(6″-feruloylglucoside), vitexin, isovitexin, astragalin and 4,7-dihydroxy-2H-1-benzopyran-2-one (peak z = 1.75). In contrast, stem (mean z = −0.297, median z = −0.545), young shoot (mean z = −0.284) and rhizome (mean z = −0.209) showed overall depletion of this super-pathway, each retaining only a narrow set of elevated compounds: L-tyrosine (z = 1.436), spermidine and sinapine in stem; methyl eugenol (z = 1.532), caffeic acid and p-coumaryl alcohol 4-glucoside in young shoot; and trans-4-coumaric acid (z = 1.586), cinnamic acid, ferulic acid, 8-gingerol and 4′,7-dihydroxyflavanone in rhizome. Thus, at the level of steady-state pool sizes, phenylpropanoid-derived metabolites are not uniformly depleted underground: rhizome and young shoot accumulate predominantly hydroxycinnamic acids, whereas root accumulates flavan-3-ols/isoflavones and leaf accumulates flavone/flavonol glycosides. Whether these accumulation patterns reflect differences in biosynthetic flux, transport or turnover cannot be resolved from metabolite pools alone.

### 3.6. Organ Distribution of Pharmacopoeial Quality Control Markers and Pathway-Level Activity Flow

**Figure 6:**
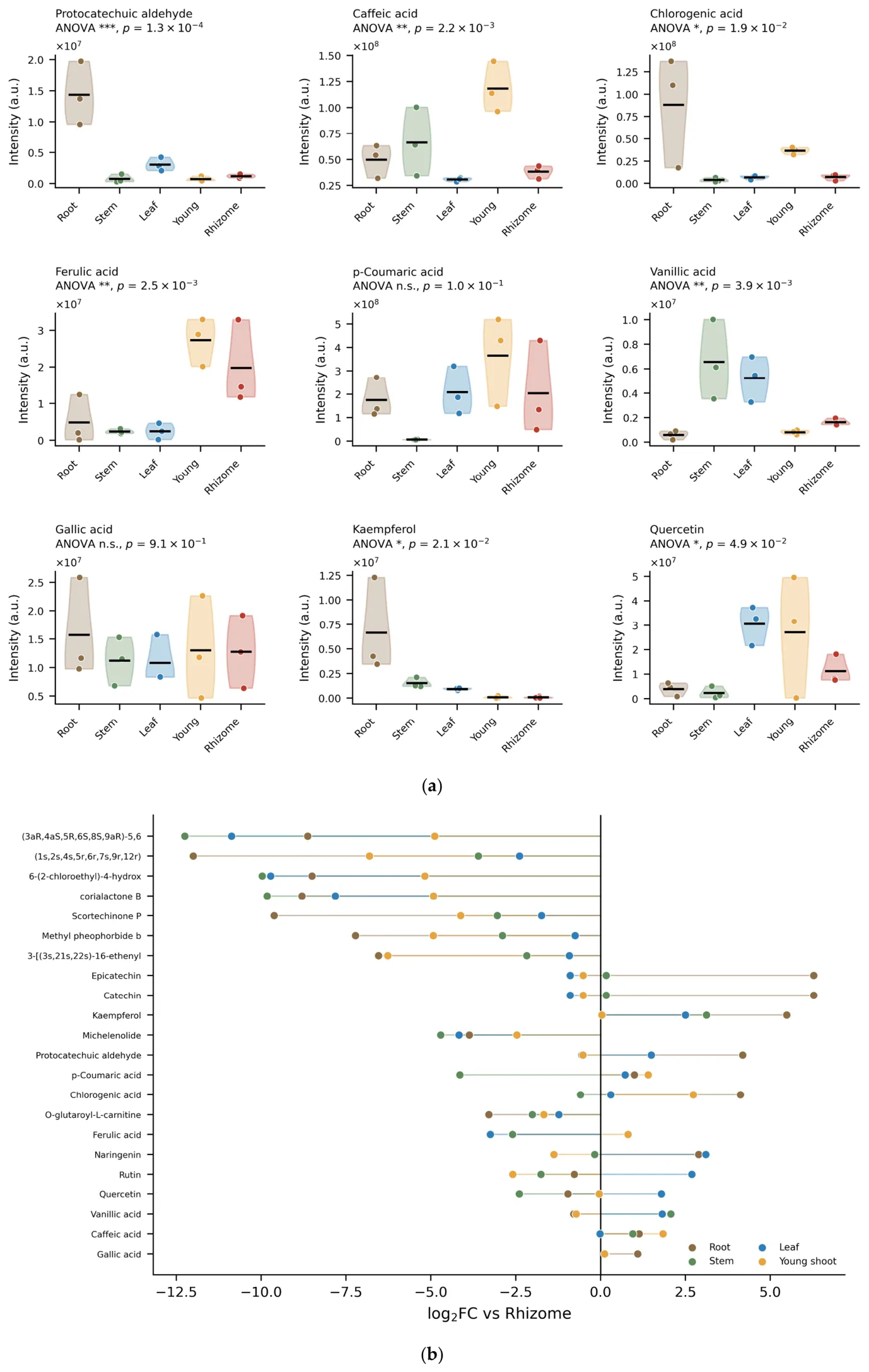

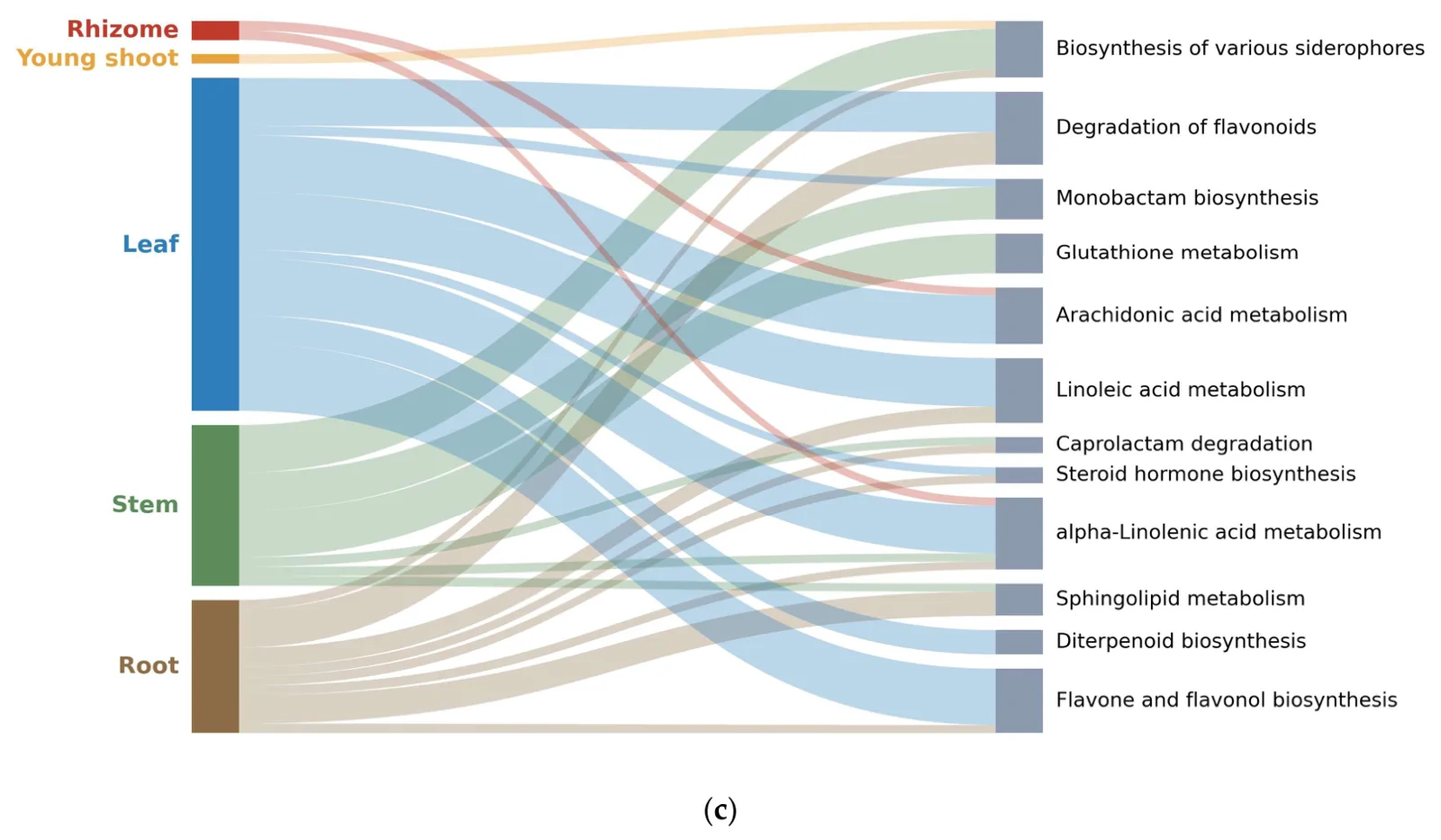
Organ distribution of quality control markers and pathway activity flow. (**a**) Violin/dot plots of absolute intensities (×10^6^) for nine representative pharmacopoeial markers across the five organs (*n* = 3 biological replicates per organ; points, individual replicates; horizontal bar, group mean). One-way ANOVA *p* values are shown above each panel (* *p* < 0.05, ** *p* < 0.01, *** *p* < 0.001, ns, not significant). (**b**) Dumbbell plot of log_2_ fold change relative to rhizome (WP) for the 22 matched marker metabolites; each point represents one organ (root, stem, leaf, young shoot) and the vertical line marks log_2_FC = 0 (rhizome reference). Metabolites are ordered by the magnitude of their maximal deviation. (**c**) Sankey diagram linking organ-specific metabolites (Tau > 0.6) to the 12 most active KEGG pathways (≥5 detected member metabolites; activity defined as the organ-mean member z-score). Left nodes, source organs; right nodes, pathways; ribbon width is proportional to the number of organ-specific metabolites mapped to each pathway.

Twenty-two pharmacopoeial or candidate quality control markers were matched within the dataset and profiled across organs (Figure 6a,b). Each organ was characterised by a distinct dominant marker: gallic acid in root (z = 1.776), michelenolide in rhizome (z = 1.608), rutin in leaf (z = 1.567), caffeic acid in young shoot (z = 1.336) and vanillic acid in stem (z = 1.156). One-way ANOVA across the five organs for nine representative phenolic markers showed significant organ effects for 7 of 9 compounds: protocatechuic aldehyde (F = 18.35, *p* = 1.34 × 10^−4^; highest in root), caffeic acid (F = 9.20, *p* = 2.20 × 10^−3^; young shoot), ferulic acid (F = 8.86, *p* = 2.53 × 10^−3^; young shoot), vanillic acid (F = 7.86, *p* = 3.92 × 10^−3^; stem), chlorogenic acid (F = 4.89, *p* = 1.91 × 10^−2^; root), kaempferol (F = 4.70, *p=* 2.15 × 10^−2^; root) and quercetin (F = 3.50, *p* = 4.91 × 10^−2^; leaf), whereas p-coumaric acid (F = 2.57, *p* = 0.103) and gallic acid (F = 0.24, *p* = 0.909) were distributed evenly among organs and are therefore poor discriminators of plant part.

Using the rhizome (WP), the officinal part, as reference, log_2_ fold changes revealed that 13 of the 22 markers were more abundant in at least one non-rhizome organ, whereas only 9 were maximal in the rhizome itself (Figure 6b). Classification by dominant organ assigned nine markers to rhizome, six to root, three each to leaf and young shoot and one to stem. The rhizome-restricted group consisted almost exclusively of terpenoid/lactone constituents that were depleted by up to three orders of magnitude elsewhere, the extreme case being (3aR,4aS,5R,6S,8S,9aR)-5,6-dihydro derivative (log_2_FC = −12.25 in stem), together with (1s,2s,4s,5r,6r,7s,9r,12r), 6-(2-chloroethyl)-4-hydroxy derivative, corialactone B, scortechinone P, methyl pheophorbide b and michelenolide. By contrast, the phenolic and flavonoid markers were strongly enriched outside the rhizome: catechin and epicatechin were >6 log_2_ units higher in root, kaempferol +5.5 (root), protocatechuic aldehyde +4.2 (root), chlorogenic acid +4.1 (root) and +2.7 (young shoot), naringenin +3.1 (leaf), rutin +2.7 (leaf) and quercetin +1.8 (leaf). These results indicate that the non-officinal organs, particularly root and leaf, are not chemically redundant but constitute quantitatively superior sources of several regulated phenolic markers, whereas the terpenoid lactone signature remains a rhizome-exclusive authentication criterion.

Finally, pathway activity was estimated for the 155 KEGG pathways containing ≥5 detected member metabolites by averaging member z-scores per organ (Figure 6c). The 12 most active pathways and their peak organs were flavone and flavonol biosynthesis (peak z = 1.453, leaf), diterpenoid biosynthesis (1.096, leaf), sphingolipid metabolism (1.046, root), α-linolenic acid metabolism (1.007, leaf), steroid hormone biosynthesis (0.999, root), caprolactam degradation (0.995, root), linoleic acid metabolism (0.967, leaf), arachidonic acid metabolism (0.964, leaf), glutathione metabolism (0.963, stem), monobactam biosynthesis (0.938, stem), degradation of flavonoids (0.911, leaf) and biosynthesis of various siderophores (0.898, stem). Mapping the 2031 tissue-specific metabolites (Tau > 0.6) onto these pathways produced a Sankey network in which leaf was by far the largest source of specific metabolite–pathway links (35 mappings), followed by root and stem, while young shoot and rhizome contributed only marginal flows. α-Linolenic acid metabolism received the most links overall (*n* = 9). Each organ channelled its specific metabolites into a characteristic destination: root → degradation of flavonoids (*n* = 4), stem → glutathione metabolism (*n* = 5), leaf → flavone and flavonol biosynthesis (*n* = 7), young shoot → biosynthesis of various siderophores (*n* = 1) and rhizome → α-linolenic acid metabolism (*n* = 1). This flow structure recapitulates the marker-level findings, assigning flavonoid biosynthesis to the leaf, flavonoid turnover and sterol/sphingolipid metabolism to the root, and redox/glutathione-centred metabolism to the stem.

## 4. Discussion

The present study provides the first organ-resolved metabolic atlas of *Cibotium barometz*, a tree fern whose rhizome (Gouji) has been listed as an officinal drug for centuries while its root, stem, leaf and young shoot are routinely discarded as processing residues. By profiling 2765 annotated metabolites across five organs, we show that this species partitions its chemistry with a precision that is neither predicted by the above-/below-ground dichotomy nor captured by the current single-organ pharmacopoeial framework. Two conclusions emerge that bear directly on the pharmacognostic status of the species: the rhizome is chemically distinctive, but its distinctiveness resides in a narrow terpenoid–lactone signature rather than in a general superiority of medicinal chemistry; and the non-officinal organs, particularly root and leaf, are quantitatively richer sources of several of the very phenolic markers by which the drug is currently assayed.

The higher within-organ CVs of root, rhizome and young shoot (median 51.7–56.1%) compared with leaf and stem (32.5–41.8%) deserve emphasis, because they directly affect the confidence that can be placed in single-metabolite differences involving these organs. Part of this dispersion is attributable to the pooling design, in which each replicate integrates several plants whose underground and meristematic tissues may have differed in age, cellular composition and developmental stage. We propose, as a hypothesis that was not tested here, that the heterogeneous cellular composition and steep developmental gradients of these organs contribute to the observed variance; single-cell or spatially resolved analyses would be needed to test this. Practically, high CVs reduce statistical power and inflate the uncertainty of fold-change estimates: an organ-specific claim for any individual metabolite in root, rhizome or young shoot should be considered provisional unless it is supported simultaneously by PCA/DAM analysis, an FDR-controlled test and a high Tau value, and even then, should be confirmed by targeted quantification.

Comparable organ-dependent differentiation has been reported in *Polygonum cuspidatum*, in which roots and rhizomes were enriched in stilbenes and anthraquinones, whereas leaves and flowers contained more flavonoids [22], and in *Kadsura coccinea*, where roots, stems and leaves possessed both shared and organ-specific metabolites [21]. Widely targeted profiling of five organs of *Camellia luteoflora* likewise identified leaves as the organ containing the largest repertoire of dominant metabolites [19]. At a finer spatial scale, mass-spectrometry imaging of *Pueraria* roots has shown that pharmacologically relevant isoflavonoids can be differentially allocated between the xylem and phloem [33]. The present results extend these observations to a medicinal tree fern and show that extensive organ specialisation was already established in an early-diverging vascular plant lineage.

Root-specific metabolites additionally showed the highest median Tau of any organ (0.866), implying that root-restricted compounds are the most exclusively partitioned, whereas stem and young shoot combined the smallest pools (*n* = 161 and 103) with the lowest median Tau (0.771 and 0.743), a signature compatible with organs that function largely as conduits whose chemistry overlaps with adjacent tissues. This root–leaf polarity is biologically plausible. Leaves experience solar radiation, oxidative load and herbivory, and characteristically accumulate flavone and flavonol glycosides that contribute to photoprotection, redox buffering and vacuolar storage; increased accumulation of flavone *O*-glycosides has been shown experimentally to enhance UV-B tolerance in rice [34]. This provides a functional context for the preferential accumulation of luteolin derivatives, vitexin, isovitexin, astragalin and rutin in *C. barometz* leaves. By contrast, the root was characterised by catechin, epicatechin, chlorogenic acid, daidzein and related intermediates. The complementary accumulation of flavan-3-ols/isoflavones in root, flavone/flavonol glycosides in leaf and hydroxycinnamic acids in rhizome and young shoot is consistent with, but does not demonstrate, organ-specific expression or activity of branch-point enzymes such as CHS, F3H, DFR/LAR and IFS, as reported for other species [35]. Although such functions remain to be tested in *C. barometz*, the root-enriched flavan-3-ol profile is consistent with a defensive chemical barrier at the plant–soil interface [36].

The Sankey mapping of the 2031 tissue-specific metabolites reinforced this division of labour, channelling leaf-specific compounds into flavone and flavonol biosynthesis (*n* = 7), root-specific compounds into flavonoid degradation (*n* = 4) and stem-specific compounds into glutathione metabolism (*n* = 5). Such partitioning is most parsimoniously explained by organ-dependent expression of branch-point enzymes, glycosyltransferases and transporters, together with differences in vacuolar sequestration and turnover, and is compatible with transcriptomic evidence for tissue-dependent expression of flavonoid- and lignin-related genes in *C. barometz* and for enhanced flavonoid accumulation in mature rhizomes [13,14]. The relatively low flavonoid abundance observed in the rhizomes analysed here therefore does not necessarily conflict with previous findings, because rhizome age, sampling position, cultivation environment and processing stage can substantially alter composition. Indeed, the developmental study reported clear shifts from phenolic-acid enrichment in younger rhizomes to flavonoid enrichment in mature material. The rhizome-restricted terpenoid lactones may contribute to the pharmacognostic distinctiveness of *Cibotii rhizoma*, and the root- and leaf-enriched flavan-3-ols and flavonol glycosides may be responsible for part of the antioxidant and anti-inflammatory activities reported for rhizome extracts [23]. However, no bioactivity was measured in this study, and the association between organ-specific metabolites and biological effects remains untested; in vitro assays relevant to the traditional indications (osteoclastogenesis, osteoblast function, chondroprotection, anti-inflammatory and antimicrobial activity) are required before any such link can be asserted.

A central aim of this study was to ask whether the metabolome offers a chemical rationale for the official status of the rhizome. The answer that emerges is qualitative rather than quantitative. The rhizome was neither the richest organ in organ-specific metabolites (*n* = 293, third of five) nor the richest in phenolics (mean pathway z = −0.209), and 13 of the 22 matched pharmacopoeial or candidate quality control markers were more abundant in at least one non-rhizome organ. This agrees with transcriptomic evidence that three functionally characterised *C. barometz* triterpene synthases are highly expressed in the rhizome relative to the leaf [14]. The rhizome is also the organ from which cibotiumbarosides and cibotiglycerol with anti-osteoclast activity have been isolated [7], and rhizome extracts have reduced ovariectomy-induced bone loss in rats [8]. Rhizome polysaccharides have additionally promoted osteoblast differentiation through BMP2/SMAD1 signalling [37], while a water extract showed chondroprotective effects associated with suppression of NF-κB-dependent inflammatory and matrix-degradation pathways [38]. Among these studies, they suggest that the medicinal identity of *Cibotii rhizoma* may depend on a multi-component matrix that includes terpenoid-derived compounds, phenolics, glycoglycerolipids and polysaccharides rather than on the abundance of any single flavonoid [1,2,13].

Importantly, chemical distinctiveness should not be equated with demonstrated therapeutic superiority. The current LC–MS/MS dataset provides relative abundance rather than absolute dose, bioavailability or pharmacological efficacy, and it does not cover high-molecular-weight polysaccharides. Moreover, the Chinese Pharmacopoeia has historically used protocatechuic acid as the quantitative marker for *Cibotii rhizoma*, whereas most of the other compounds evaluated here are more appropriately described as reported or candidate markers rather than pharmacopoeial markers [3,39]. The proposed rhizome-specific lactones should therefore be validated with authentic standards, including matching retention times and diagnostic MS/MS spectra, before they are advanced as authentication markers. This is especially important for structurally similar terpenoids, for which library-level annotation may not distinguish stereoisomers or positional isomers. Metabolite identification confidence should be explicitly reported according to Metabolomics Standards Initiative criteria [40].

Several limitations constrain the generality of our findings. First, all plants were collected from a single cultivation site in a single month; the Tau-based organ-specific repertoires therefore describe one population at one developmental time point, and their consistency across geographical origins, seasons, plant ages and genotypes is unknown. Independent validation with material from other provenances and harvest times is a necessary next step, and we regard the fingerprints reported here as candidates rather than validated authentication markers. Second, the three replicates per organ are pools, so inter-individual variability was not estimated. Third, most annotations, including the rhizome-associated terpenoid lactones, are putative (MSI level 2–3) and require confirmation with authentic standards. Fourth, the data are relative peak areas from a methanol/acetonitrile/water (2:2:1, *v*/*v*/*v*) extract and do not capture non-polar lipids or volatiles; “enrichment” throughout refers to relative, not absolute, abundance. Finally, no pathway reached FDR < 0.05 in enrichment testing, and all pathway-level interpretations are exploratory.

## 5. Conclusions

*C. barometz* possesses a highly compartmentalised metabolome in which the rhizome retains a distinctive candidate terpenoid/lactone signature, while root and leaf contain larger and chemically complementary phenolic repertoires. These findings support the chemical authentication of the rhizome but do not imply that it contains all potentially valuable constituents of the plant. Integrating organ-resolved metabolomics with quantitative chemistry, transcriptomics, pharmacological validation and sustainable harvesting experiments should enable both more specific quality control of *Cibotii rhizoma* and responsible utilisation of selected non-officinal biomass.

## Data Availability

The processed metabolomics data and the associated metabolite annotation metadata supporting this study are available in Figshare at doi: https://doi.org/10.6084/m9.figshare.33295689. The deposited annotation table is the single alignment described in the Provenance of the Data and Relationship to the Originally Submitted Method Description section and contains the root (WR), stem (WS), leaf (WL), young shoot (WN) and rhizome (WP) sample groups analysed here together with the field-domesticated (DM) and tissue-cultured (ZP) rhizome groups of the companion study [23] and the four pooled QC injections; the data relevant to the present manuscript comprise the WR, WS, WL, WN and WP groups. The native Thermo instrument files (.raw) for all injections, including the QC and solvent-blank injections of both polarities, the Compound Discoverer 3.3 result files (.cdResult) and the instrument acquisition methods have been deposited in the same repository and are available to the editors and reviewers on request, so that the acquisition platform and the reported feature table can be verified directly at source.

## Supplementary Materials

The following supporting information can be downloaded at https://www.mdpi.com/article/10.3390/metabo16090693/s1: Table S1: The complete list of included metabolites for 15 samples of *C. barometz*. Table S2. Quantification and chemical annotation of metabolites identified across the five sample groups. Table S3. Platform-consistency and provenance audit of the deposited feature table, reporting feature and annotation counts, the adduct inventory, the measured-versus-theoretical mass-accuracy distribution, the retention-time distribution, per-feature QC RSD, the QC pool-composition test and the batch-linkage test against the companion rhizome dataset. Figure S1. Total ion chromatograms (TICs) of four pooled quality control (QC) injections analysed in positive ionisation mode, displayed as an overlay. The individual traces correspond to QC injections interspersed throughout the analytical sequence (QC1–QC4), with the value following each QC label denoting the total ion count applied for normalisation of that trace. Figure S2. Overlay of the TICs obtained from the same four pooled QC injections under negative ionisation conditions. Display format and normalisation follow those detailed in Figure S1.
